# Preheating 2-Octyl Cyanoacrylate Reduces Curing Time in Robotic Total Knee Arthroplasty: A Pilot Randomized Study

**DOI:** 10.7759/cureus.88189

**Published:** 2025-07-17

**Authors:** Ren Yi Kow, Woon Theng Lo, Yong Ng, Jeremy Tze En Lim, Chun Lei Tan, Ming Hua Jonathan Cheng, Ming Han Lincoln Liow

**Affiliations:** 1 Department of Orthopaedics, Traumatology & Rehabilitation, International Islamic University Malaysia, Kuantan, MYS; 2 Department of Orthopaedic Surgery, Singapore General Hospital, Singapore, SGP; 3 Department of Anaesthesiology, Singapore General Hospital, Singapore, SGP

**Keywords:** quality improvement research, robotic-assisted total knee arthroplasty, tissue adhesive, tka, wound closure technique

## Abstract

Introduction

Tissue adhesives, such as 2-octyl cyanoacrylate, are widely used for tissue approximation and protection of surgical wounds during total knee arthroplasty (TKA). However, drying time for tissue adhesives can increase overall operating time and impede efficiency. Various methods may reduce the drying time of tissue adhesives. However, there is currently no clinical or in vivo data to support these findings. This study aims to investigate whether preheated 2-octyl cyanoacrylate (Dermabond™, Ethicon, Inc., Somerville, NJ, US) shortens drying time in robotic-assisted TKA.

Methods

This randomized pilot study included 40 patients undergoing TKA, and they were randomized into two groups: the intervention group received Dermabond™ preheated in a solution warmer device, while the control group received Dermabond™ at ambient temperature. Incision size, preparation, and drying times were recorded and compared. Comparison of drying time, preparation time, incision size, BMI, weight, height, age, surgery time, and gender between the preheated and control groups was conducted using the t-test, Levene’s test, and the chi-squared test.

Results

Data from 39 patients were analyzed, as one intervention failed due to premature Dermabond™ drying in the preheated group. The drying time was reduced by an average of 26.8 seconds in the preheated group compared to the control group (p < 0.05). No significant differences were observed in preparation time or incision size between the groups.

Conclusion

Preheating Dermabond™ reduces drying time; however, the reduction does not provide substantial time savings to alter the current TKA workflow. Improvements in intraoperative efficiency may be better achieved by optimizing other aspects of the procedure.

## Introduction

Total knee arthroplasty (TKA) is a highly effective surgery for patients with severe knee osteoarthritis [1]. Robotic TKA has shown potential for increased surgical accuracy and improved outcomes compared to conventional TKA [2,3]. Despite advances in surgical techniques, wound closure remains a crucial component of optimizing patient outcomes, minimizing complications, and improving intraoperative efficiency [4,5]. Tissue adhesives, particularly 2-octyl cyanoacrylate (Dermabond™, Ethicon, Inc., Somerville, NJ, US ), have emerged as a viable alternative or adjunct to traditional suturing techniques due to their ability to create a protective barrier against microbial infiltration and reduce wound-related complications [6,7].

While the benefits of tissue adhesives in wound closure have been well documented, one challenge remains: the drying time of tissue adhesives. The polymerization of Dermabond™ is an exothermic reaction, and the delay in drying time can lead to an operative standstill, affecting operating room (OR) efficiency. Various in vitro strategies have been described to accelerate the drying process, including covering the wound, using OR lights, and employing hot water baths [8]. Among these, preheating the adhesive before application has been proposed as a potential method to expedite curing time. However, there is currently no clinical in vivo data to support these findings.

In musculoskeletal oncology and orthopedic surgeries, studies have investigated the application and outcomes of Dermabond™, reporting high patient satisfaction and low complication rates [9-12]. However, research specifically examining the effects of prewarming Dermabond™ on drying time in robotic TKA remains limited [13]. Therefore, this study aims to investigate whether preheated 2-octyl cyanoacrylate (Dermabond™) shortens drying time in robotic-assisted TKA.

## Materials and methods

This was a pilot randomized study of patients undergoing TKA from November 2024 to December 2024. The following inclusion criteria were applied: (1) robotic-assisted TKA under the same consultant arthroplasty surgeon, (2) TKA performed for knee osteoarthritis, and (3) similar anterior midline incision and medial parapatellar approach. Exclusion criteria were as follows: (1) TKA performed for secondary arthritis such as rheumatoid arthritis, (2) TKA performed under no tourniquet technique, (3) allergy to tissue adhesive, and (4) wound requiring vacuum-assisted closure (VAC).

**Figure 1:**
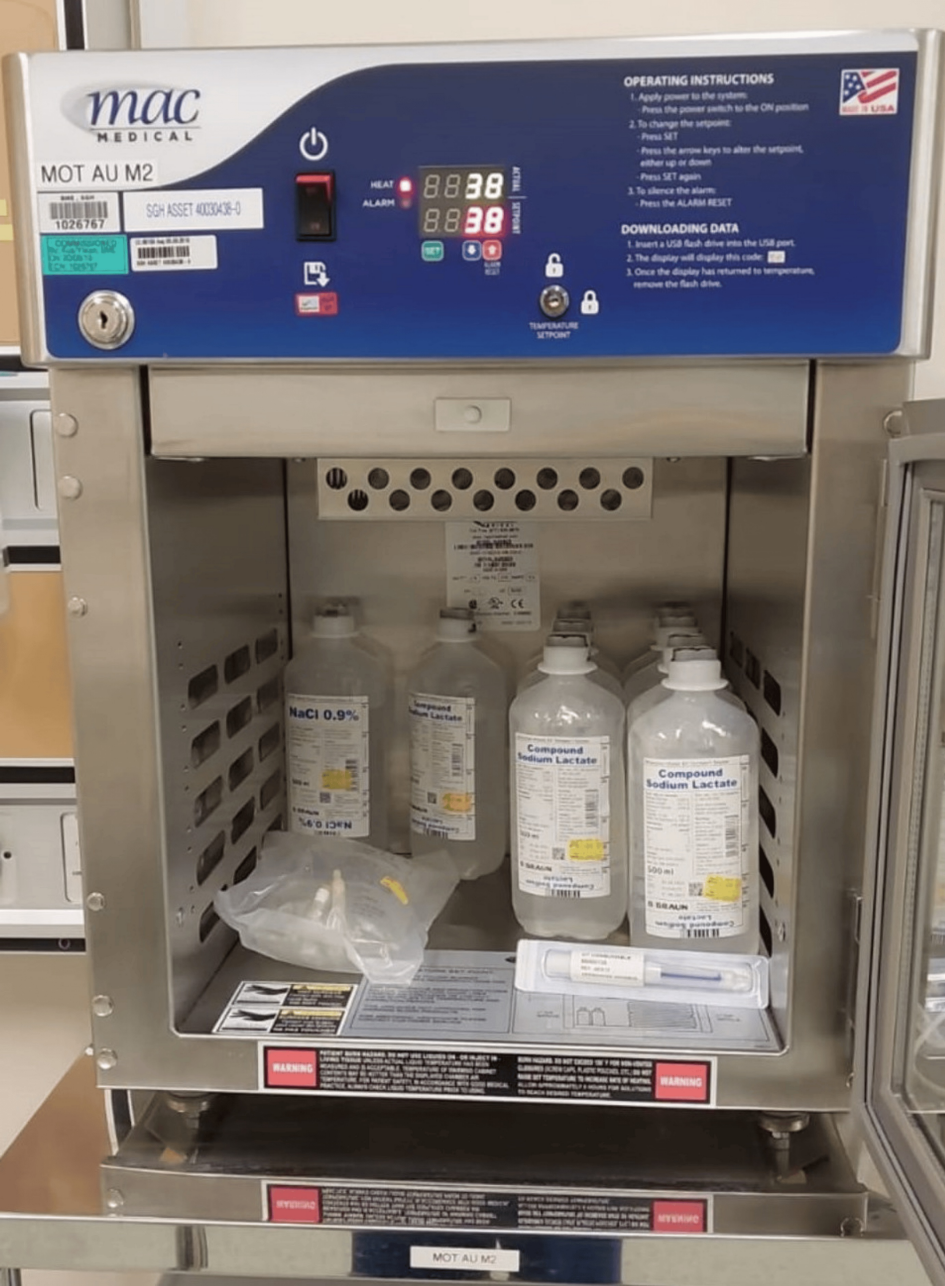
The Dermabond™ was kept in a solution warmer device set at 38°C. It was removed from the warmer device and opened prior to the closure of the skin.

All the patients underwent TKA under similar techniques and conditions. After the implantation of the knee prostheses, cocktail injections were given with a mixture of 1.5 g tranexamic acid prior to joint capsule closure. The joint capsule was closed with Stratafix™ 1 (Ethicon, Inc.), and the skin was closed with Stratafix™ 3 by two residents. No suture material was applied to the subcuticular tissue layer. The allocations were done prior to the case starting, where the Dermabond™ was either kept in the warmer (prewarmed group) or kept in the sterile tray (control group). The Dermabond™ was kept in a solution warmer device set at 38°C (Figure 1). The prewarmed Dermabond™ was opened upon the closure of the skin. Two fixed residents applied the tissue adhesive evenly on the skin surface, and the preparation was timed from the applicator activation to even distribution on the patient skin. The drying time was assessed manually by testing on the edges of the wound until complete dryness. All the patients were given Convatec Aquacel^®^ Ag (Deeside, UK) dressing after the Dermabond™ dried.

In terms of sample size calculation, data from Wellington et al. served as the basis for the calculation [8]. Their data were normally distributed, with a standard deviation (SD) of 28 seconds. The true difference between the intervention and control groups was shown to be 30 seconds. Based on this data, in order to demonstrate a difference between the intervention and control groups with a 1:1 allocation ratio, a total of 15 intervention subjects and 15 control subjects were required. Taking into account a 25% dropout rate or non-intervention, the minimum number of participants needed was 38. Hence, we proceeded to recruit 40 patients for this prospective study.

Statistical analysis was performed using SPSS software version 20 (IBM Corp., Armonk, NY, US). Comparison of drying time, preparation time, incision size, BMI, weight, height, age, surgery time, and gender between the preheated and control groups was conducted using the t-test, Levene’s test, and the chi-squared test. The mean and SD were used in the analysis instead of the median and interquartile range (IQR) because the Shapiro-Wilk test demonstrated that data for both groups were normally distributed. The primary focus of the study was to determine the difference in curing time between the two groups, and the skewness for the preheated and control groups was 0.15 and 0.07, respectively, indicating an almost symmetric distribution of the dataset.

## Results

The study enrolled 40 patients but included 39 patients for analysis, with 19 in the preheated group (as one intervention failed due to the premature drying of Dermabond™) and 20 in the control group. Table 1 presents the demographic and clinical characteristics of the study participants. Overall, the demographic and clinical characteristics were well-balanced between the two groups, except for a slight difference in age. These findings suggest that the groups were comparable at baseline, minimizing the risk of confounding variables affecting the primary outcome.

**Table 1:** Demographics of the patients recruited. *Data shown in mean and standard deviation.

Demographic		Preheated group	Control group	p-value
Gender	Male	7	9	0.748
	Female	12	11	
Side	Left	13	7	0.056
	Right	6	13	
Age (years)*		70.11 (±6.31)	73.65 (±4.11)	0.043
Height (cm)*		155.47 (±11.41)	158.35 (±9.43)	0.395
Weight (kg)*		66.98 (±16.04)	67.23 (±13.35)	0.96
BMI (kg/m^2^)*		27.50 (±5.25)	26.69 (±3.94)	0.59
Total		19	20	

Table 2 summarizes the surgical factors. Notably, the drying time was significantly reduced in the preheated group, with a mean difference of 26.85 seconds compared to the control group (116.05 ± 32.21 s vs. 142.90 ± 39.05 s, p = 0.025). However, no statistically significant differences were observed in preparation time, incision size, BMI, weight, height, or total surgery time between the two groups. The preparation time was similar (26.58 ± 2.91 s vs. 26.05 ± 1.93 s, p = 0.506), as was the incision size (15.66 ± 1.45 cm vs. 15.60 ± 1.24 cm, p = 0.894). Mean total surgery time was also comparable (64.21 ± 16.01 min vs. 66.25 ± 19.05 min, p = 0.720). All patients recovered well with no wound complications.

**Table 2:** Surgical factors comparing the preheat group and the control group. Data shown in mean and standard deviation. **The Shapiro-Wilk test demonstrated that data for the preheated and control groups were 0.15 and 0.07, respectively, indicating a normally distributed dataset.

Surgical factors	Preheated group (n = 19)	Control group (n = 20)	p-value
Incision size (cm)	15.66 (±1.45)	15.60 (±1.24)	0.894
Preparation time (s)	26.58 (±2.91)	26.05 (±1.93)	0.506
Drying time (s)**	116.05 (±32.21)	142.90 (±39.05)	0.025
Total surgery time (min)	64.21 (±16.01)	66.25 (±19.05)	0.720

## Discussion

Wound closure techniques play a critical role in postoperative healing, infection prevention, and surgical efficiency in TKA. Traditional closure methods, including sutures and staples, have been widely used, but they present certain drawbacks such as higher infection rates, poor cosmetic outcomes, and the need for dressing changes [14-19]. As an alternative, tissue adhesives such as Dermabond™ have gained popularity due to their ability to create a strong, flexible barrier that reduces bacterial contamination, eliminates the need for suture removal, and allows for quicker wound closure [6,7,15]. Moreover, studies on tissue adhesives in TKA also suggest benefits such as reduced dressing changes and improved patient satisfaction [10,16].

Of particular interest is Dermabond™’s drying time, which can contribute to delays in operative workflow. Prior research suggests that preheating can reduce Dermabond™ drying time, but these studies were laboratory-based (in vitro) or applied to other surgical contexts [8,12]. Despite numerous studies having examined wound closure efficiency, cosmetic outcomes, and infection prevention, there remain limited clinical data on optimizing tissue adhesive drying time especially in robotic TKA [14-16]. Our study adds to this body of evidence by providing clinical data on the role of prewarming in a real-world surgical setting.

Prior to this prospective randomized study, a pilot study was conducted to investigate the feasibility of various drying methods used to accelerate the drying process of Dermabond™ [8]. With the study by Wellington et al. serving as the backdrop for this research, we examined the feasibility of all methods performed in their study [8]. As both the fanning and UV light methods had been shown to be inefficient in reducing curing time, they were not considered for this prospective study. Despite covering the area shown to reduce curing time, this method was not feasible in real-world settings, as maintaining sterility while covering the entire 15 cm TKA wound was challenging. Similarly, although using the OR lights reduced drying time, it was not feasible in clinical settings, as sterility could be compromised when bringing the OR lights closer to the wound. Additionally, different ORs have varying numbers of OR lights, and the distance between the light and the wound would be difficult to quantify accurately, making standardization difficult.

The hot water bath showed promising results in our pilot study. Nevertheless, we noted that warming the Dermabond™ with hot water was difficult to standardize, as the duration of the surgery could affect the temperature of the water used for soaking the Dermabond™. To circumvent this obstacle, we used a solution-warming device, as the temperature used to warm the solution was similar to that of hot water, and the Dermabond™ could be opened whenever skin closure was completed.

The demographic and clinical characteristics of the study population were generally well-matched between the preheated and control groups, minimizing potential confounding factors. Gender distribution was similar, and there were no significant differences in BMI, weight, or height, suggesting that body composition did not influence the outcomes of this study. While there was a slight variation in the laterality of the surgical site, this difference was not statistically significant. Notably, the control group had a slightly higher mean age than the preheated group (73.65 ± 4.11 years vs. 70.11 ± 6.31 years, p = 0.043). Although this difference reached statistical significance, it is unlikely to have had a meaningful impact on the primary outcome. Overall, the well-balanced baseline characteristics indicate that any observed differences in drying time were likely due to the intervention rather than demographic variations.

This study demonstrated that prewarmed Dermabond™ significantly reduced drying time by an average of 26.85 seconds compared to the control group (p = 0.025). However, no significant differences were observed in preparation time, incision size, BMI, weight, height, or total surgery time, indicating that preheating primarily affects curing time without impacting other surgical parameters. This finding aligns with the study by Wellington et al., who explored various methods to accelerate drying time and identified preheating as a promising technique [8]. Despite the statistical significance of this reduction, the clinical impact on intraoperative workflow remains minimal. Additionally, an important consideration is that the manufacturer recommends storing Dermabond™ at temperatures not exceeding 30°C, whereas the preheated group used a temperature of 38°C [20]. This deviation from the recommended storage conditions may alter the adhesive’s properties, potentially influencing its bonding strength and durability. Nevertheless, all patients in this study recovered fully without any wound complications, suggesting that the intervention did not compromise wound healing.

Our study has limitations that warrant consideration. Firstly, the study included only 40 patients, which limits the generalizability of the results. Nevertheless, despite the small sample size, this study demonstrated a significant difference between the two groups. Secondly, while all the patients in this study recovered well with no wound complications, the small sample size may underestimate the negative effects of prewarming, as excessive heat exposure may alter the adhesive’s properties, potentially affecting bonding strength and the durability of wound closure. Future material integrity testing can be conducted to determine the optimal prewarming temperature to ensure the adhesive's efficacy and safety. Thirdly, we did not investigate the temperature of the skin surface. Since the temperature of the patients’ skin surface may affect the curing time of Dermabond™, it could potentially be a confounding factor. In view of the issues raised, further research is needed to assess long-term outcomes, standardize drying time measurement, and broaden applicability in different surgical settings. The clinical significance of this time reduction should be further investigated, particularly in high-volume surgical centers where cumulative time savings could impact efficiency.

## Conclusions

Preheating Dermabond™ reduces curing time; however, the reduction is not substantial enough to alter the current TKA workflow. Preheating may contribute to marginal improvements in intraoperative efficiency, but its adoption in standard practice will depend on further research assessing patient outcomes, cost-effectiveness, and surgical workflow benefits. Improvements in intraoperative efficiency may be better achieved by optimizing other aspects of the procedure.
